# Enhanced Physicochemical Stability of the L-DOPA Extract of *Mucuna pruriens* Seeds by Adding *Phyllanthus emblica*

**DOI:** 10.3390/molecules28041573

**Published:** 2023-02-06

**Authors:** Chayarit Vilairat, Khwanlada Kobtrakul, Sornkanok Vimolmangkang

**Affiliations:** 1 Graduate Program in Pharmaceutical Science and Technology, Faculty of Pharmaceutical Sciences, Chulalongkorn University, Bangkok 10330, Thailand; 2 Center of Excellence in Plant-Produced Pharmaceuticals, Faculty of Pharmaceutical Sciences, Chulalongkorn University, Bangkok 10330, Thailand; 3 Department of Pharmacognosy and Pharmaceutical Botany, Faculty of Pharmaceutical Sciences, Chulalongkorn University, Bangkok 10330, Thailand

**Keywords:** levodopa, mung bean, amla fruit, green technology, Parkinson’s disease

## Abstract

Levodopa (L-DOPA) is an essential drug for the treatment of Parkinson’s disease. Currently, L-DOPA can be produced by chemical synthesis and can also be found naturally in many herbs, especially *Mucuna Pruriens* (MP). According to clinical research, the MP extract containing L-DOPA for the treatment of Parkinson’s disease could reduce side effects more than the synthetic one. Unfortunately, MP extracts can be easily degraded. Changes in physical and chemical properties such as the appearance (color, melt, solid lump) and the reduction of L-DOPA content in the extract were commonly observed. Therefore, it is necessary to develop an extraction procedure to stabilize the extract of L-DOPA. This study attempted to enhance the extraction process by modifying the traditional acidification approach using hydrochloric acid, citric acid, or ascorbic acid. According to the stability test results, using *Phyllanthus emblica* water (PEW) as a solvent improved the preservative properties more than other solvents. The color of the PEW-MP powder changed slightly after 12 months of accelerated storage, but the amount of L-DOPA remained the highest (73.55%). Moreover, L-DOPA was only detected in MP and PEW-MP, but not PEW alone (the HPTLC chromatogram at Rf 0.48 and the HPLC chromatogram at Rt 6.0 min). The chemical profiles of PEW and L-DOPA observed in the chromatograms indicated that they are independently separated. As a result, they can be applied to a quality control process. Therefore, PEW was proven to be a powerful solvent for L-DOPA herbal extract that could be readily used as a raw material for herbal products.

## 1. Introduction

L-DOPA is a first-line drug that is used to treat a symptom of Parkinson’s disease (PD). It is produced by chemical synthesis [1]. However, the compound can be found in nature, particularly in legumes such as *Canavalia ensiformis*, *Cassia hirsute*, and *Mucuna pruriens* (MP) [2]. Synthetic L-DOPA is combined with the drug group of decarboxylase inhibitors. However, many patients experience side effects such as nausea, vomiting, and low blood pressure [3]. It was also discovered that high concentrations of L-DOPA in the bloodstream, or long-term treatment with synthetic L-DOPA, increase the risk of dyskinesia, which is a twisted movement of the limbs and trunk in which movements cannot be controlled [4]. Several studies have shown that MP can reduce the unpleasant side effects of dyskinesia in vivo [5] and reduce adverse events (nausea, dizziness, somnolence, and psychiatric) in humans [6].

The powder of MP seed has been clinically studied for PD treatment and was found to be more effective and had fewer side effects than synthetic drugs [7]. Interestingly, the appearance of dyskinesia was compromised when MP was used. It was suspected that MP might contain certain active ingredients that act as decarboxylase inhibitors [8]. However, prolonged PD treatment has a clinical effect due to motor complications. Due to the neurotoxic effect of L-DOPA in treating the disease, the administration of L-DOPA is delayed, if possible, to avoid side effects. Therefore, combining L-DOPA therapy with antioxidants was investigated to possibly reduce L-DOPA-induced oxidative stress, and it was found that the oxidative toxicity of L-DOPA was reduced significantly in mice [9]. The investigation of murine microglia and human neuroblastoma cells revealed that in addition to L-DOPA in MP, there are other compounds that reduce the cause of the nervous system as well [10]. As a result, MP containing L-DOPA is of interest as an alternative treatment and potentially better than a single synthetic drug.

**Figure 1:**
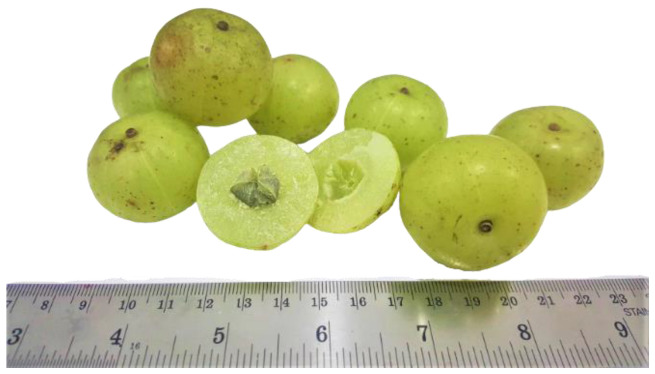
Phyllanthus emblica fruit.

Although MP extract seems to have therapeutic benefits, extraction has some limitations. Several studies are being conducted to extract L-DOPA as efficiently as possible. DOPA was extracted from MP seeds, but it was discovered that L-DOPA was easily degraded by various extraction methods such as soaking, autoclaving, roasting, sprouting, and alkaline fermentation [11]. Among all processing methods, only roasting with acid pH can increase the L-DOPA content [12]. Another interesting method is ultrasound-assisted solvent extraction, which produces a high extraction yield if used in combination with acid and heat [13]. Recently, Benfica et al., (2021) reported that aqueous solutions of citric acid showed a more effective solvent for the extraction of levodopa from MP seeds [14]. However, one of the significant limitations of L-DOPA extraction is the degradation due to the oxidative environment during the extraction process, resulting in changes in properties such as color, brownish, and the effect of atmospheric moisture [15]. Therefore, it remains necessary to find an effective extraction method. According to the above research on the treatment supplement with an antioxidant, the idea of extracting MP seeds with low acidic water from natural sources such as *Phyllanthus emblica* is of interest. *P. emblica* or Amla fruit is a medicinal plant, and its fruit has high vitamin C and other acids that make it taste sour (Figure 1). Furthermore, it has many bioactive compounds, and the animal study reported the benefits of methanolic *P. emblica* (PE) extracts, which may aid in the prevention of oxidative stress in the brain, which is a contributing factor to Parkinson’s disease and reduce the cause of Alzheimer’s disease [16,17]. In addition, the polyphenolic compounds in PE contribute to gastrointestinal protection and cardioprotective activity [18,19]. Moreover, the study also identified the benefits of PE in terms of antioxidant activity in its main bioactive compound such as gallic acid. Ascorbic acid and phenolic compounds, therefore, support the immune system and digestion [20].This study aims to enhance the physicochemical stability of MP extract containing high L-DOPA by using PEW derived from the fruit as a natural solvent. The extract would be ready to be used as a raw material in a formulation of herbal products or dietary supplements for the purpose of treatment in Parkinson’s patients and others.

## 2. Results and Discussion

### 2.1. Effect of PEW and Conventional Solvents

The extraction of MP seeds was performed to compare the effect of PEW and other conventional acid solvents. L-DOPA content was one of the main parameters used to compare the efficiency of the extraction method in this study. To quantify the amount of L-DOPA, the HPLC technique was validated and performed.

The sample extract with PEW has been proven to have extraction efficiency similar to that of hydrochloric acid (HCA). The result showed that at 2% PEW and HCA, a conventional acid solution, gave a similar yield in both extractive value and L-DOPA content. When increasing the concentration of PEW in extraction contributes to increasing the percentage of productivity. On the other hand, the amount of L-DOPA decreased because PEW extract had a very high concentration, while the proportion of dried extract consisted mainly of PEW. In comparison, the use of ascorbic acid (ASA) in the test resulted in the lowest amount of L-DOPA (3.88% *w*/*w*) due to its readily degradable solution. This is consistent with the report of Ariahu et al., (2011), who studied the kinetics of ASA loss in hot water. It was found that the percentage remain tended to decrease when heating in the range of 60–90 °C and long-time exposure effect in ascorbic content as well [21]. Other studies used ASA to stabilize L-DOPA without thermal extraction. Pappert et al., (1996) prepared levodopa solution with ascorbate and maintained it in the extraction state and found that it yielded the most stable results if stored in a refrigerator or freezer [15]. In addition, MP extracted with ethanol: water (1:1), adding a little ASA as a protector during the extraction process showed the L-DOPA content of 5.0–9.7% *w*/*w* without heating in the extraction process [22]. Considering citric acid (CTA) assisted extraction, the results were moderate. Similarly, it has been reported that CTA (58%w/v) was used to optimize the extraction process using a solid and solution ratio (1:7) and yielded an L-DOPA content of about 8%wt [23]. According to this report, there was a noticeable process difference when using a higher solid-solution ratio and a higher CTA concentration but given a similar L-DOPA content to our study. CTA may be an alternative assistant to the extraction. It has also been recently reported that CTA served as a component of a eutectic solvent (citric acid combination with cholinium chloride) by which 7.2% wt of L-DOPA was obtained [14]. However, it should be noted that such a report aimed to purify the compound and reuse the extraction solution, whereas this study aimed to develop an extract that could be used immediately without purification.

**Figure 2:**
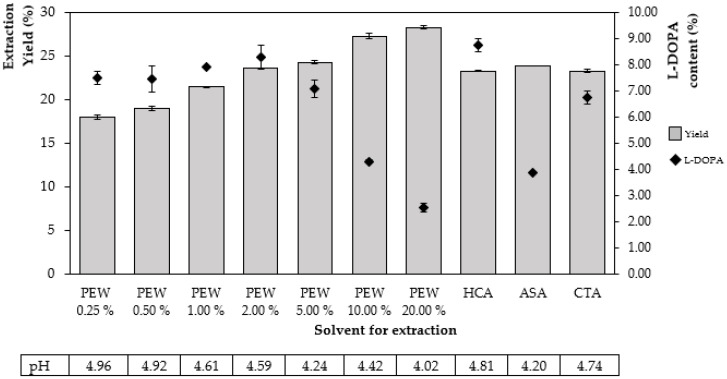
The percentage of extraction of the yield and the L-DOPA content of MP extracts with different concentrations of PEW compared to conventional solvents (HCA, ASA, and CTA). The result was reported as a mean value ± S.D. (*n* = 3).

According to the experimental results in this work, the use of PEW at a concentration of 2% was able to produce % L-DOPA similar to the aforementioned report in Misra and Wagner [22]. In this study, it has been demonstrated that various concentrations of PEW can be used as an extraction agent for L-DOPA. The results showed that the extraction yield increased significantly (Figure 2). However, a higher concentration of PEW over 5%, and a lower amount of L-DOPA was observed. This may be because the percent calculation of L-DOPA was diluted by increased extractive yield. The higher extractive value of the extracts when using a high concentration of PEW (10–20%) may be contributed by the weight of PEW powder residue after drying. The selection of the PEW concentration was then considered by the production cost and the amount of active ingredient that would be important to determine the equilibrium point in the production process.

### 2.2. Characterization of MP Chemical Profile Fingerprints Extracted with PEW

**Figure 3:**
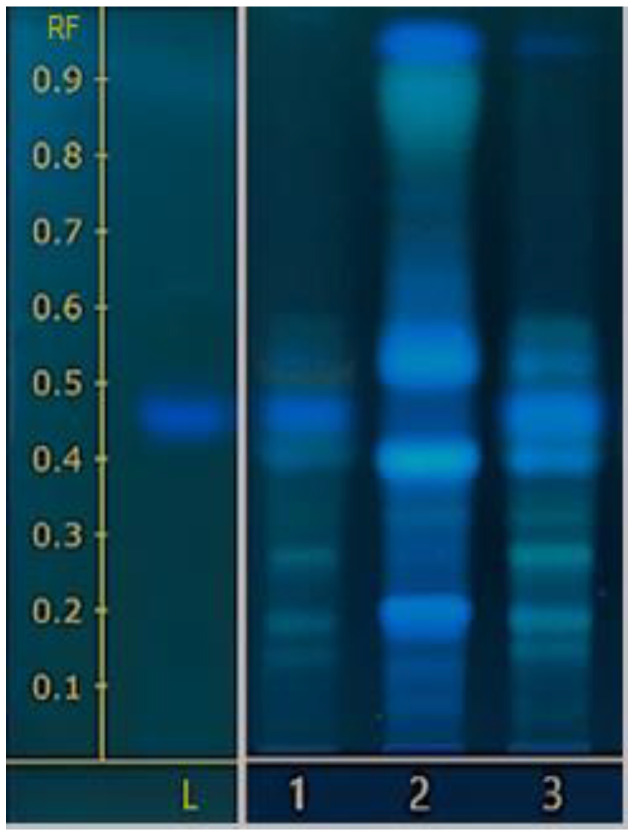
Chemical fingerprinting of the PEW-MP extract was performed by the HPTLC technique. Track (L) is the standard of L-DOPA; track 1 is the single component MP seed extract with water; track 2 is the PEW extract with water; track 3 is the extract of MP seeds extracted with PEW.

**Figure 4:**
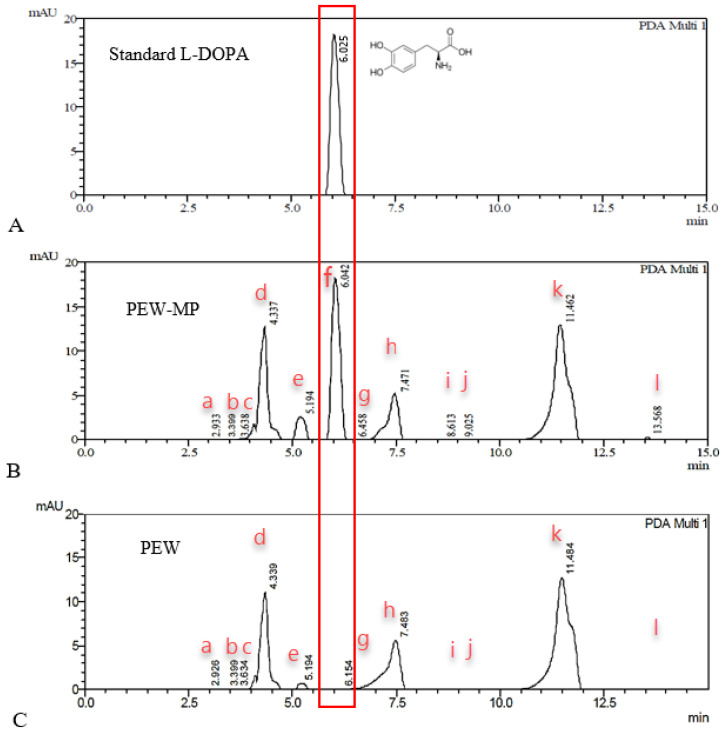
The chromatogram detected L-DOPA using the HPLC technique. (**A**) Standard L-DOPA; (**B**) PEW-MP; (**C**) PEW. The peak of L-DOPA (f) was highlighted in a red squared line at the RT around 6 min. Tailing factor (T_f_) of peak L-DOPA = 0.92; Resolution (Rs) between components e and f, and f and g = 1.8 and 2.0, respectively. Peaks a–l except f found in PEW-MP were the same as those found in PEW.

The chemical profile is important inspection quality of raw materials, ingredients, and finished products in herbal medicine [24]. HPTLC is a simple testing technique and is widely used for quality assurance of the herbs to be tested. It can be checked in conjunction with HPLC to confirm the quality of the product. HPTLC and HPLC chromatograms were documented to compare the chemical profile of PEW-MP extract, MP seed, and PEW using L-DOPA as a marker (Figure 3 and Figure 4). Figure 3 displayed the HPTLC chemical profile of standard L-DOPA (Track L), a single substance of MP seed (Track 1), PEW (Track 2), and the MP seed extracted with the PEW sample (PEW-MP) (Track 3). The chemical profile of PEW-MP was similar to that of a single substance of MP and PEW extract, and L-DOPA was also detected. Despite the fact that the PEW-MP sample was a combination of two herbs, it was proved that it could be distinguished by this technique compared to a standard sample.

Another method to ensure the presence of L-DOPA in PEW was HPLC. This method has been known to be a technique used in an assay of pharmacopeia. The development of a system to detect an active substance is necessary for the quality control of drug products. The HPLC system used in this study successfully separated and detected L-DOPA in the PEW-MP extract (Figure 4). In other words, there was no overlapping peak at the same retention time (RT) with the peak of L-DOPA at 6 min (Figure 4A). L-DOPA detected in the PEW-MP extract (Figure 4B, peak f) was independently isolated compared to a specific chromatogram of PEW by HPLC (Figure 4C). The other peaks, a–h and k–l, detected in PEW-MP were also found in PEW suggesting that they came from PEW. It implied that L-DOPA and other compounds in PEW did not interact with each other. This would maintain the value of the important substance when used in the treatment process for patients. These findings revealed the chemical profile of natural extracts, as well as the quality of the chemicals found in herbs. This research also serves as a chemical quality control to ensure the commercial authenticity of the manufacturing process. The HPLC technique has been used for detecting mixed herb extracts. For instance, the combination of *Andrographis paniculata* (Burm f.) *Ness* and *Phyllantus niruri* L. used andrographolide as a chemical marker for HPLC quantitative analysis and method validation [25]. In another study, multi-makers (gallic acid, ellagic acid, and ascorbic acid) have been used simultaneously to separate multicomponent in herbal formulations [26]. HPLC is therefore a method of choice for the determination of chemical components of interest and provides accurate results that can be applied in the quality control process.

### 2.3. Extraction of L-DOPA from Different MP Varieties by PEW

**Figure 5:**
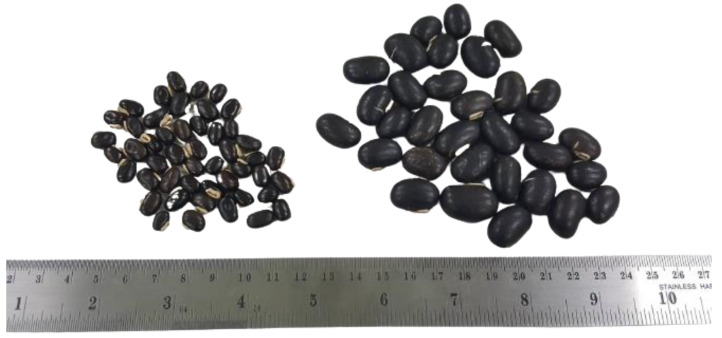
The seed characteristics of Thai and Indian *M. pruriens*. Thai MP seed (**left**) and Indian MP seed (**right**).

In the production process, raw materials may not be available as needed due to various factors. Plants similar in species or other plants can be substituted. The purpose of this experiment was to compare two main sources of L-DOPA available in Thailand, Thai MP (*M. pruriens* var. *pruriens*; MPT) and Indian MP (*M. pruriens* var. *utilis*; MPI) varieties, to prove that PEW could be used for extraction regardless of the plant variety. Both varieties differ in their character but contain L-DOPA. MPT has long and stingy hair on its seed pod, causing skin irritation when touching it [27], while MPI has short and soft hair [28]. The seed of MPT is smaller than that of MPI (Figure 5). However, MPT has an L-DOPA content of 4.91–7.09% *w*/*w*, which is comparable to MPI (3–9% *w*/*w*) [29,30].

**Figure 6:**
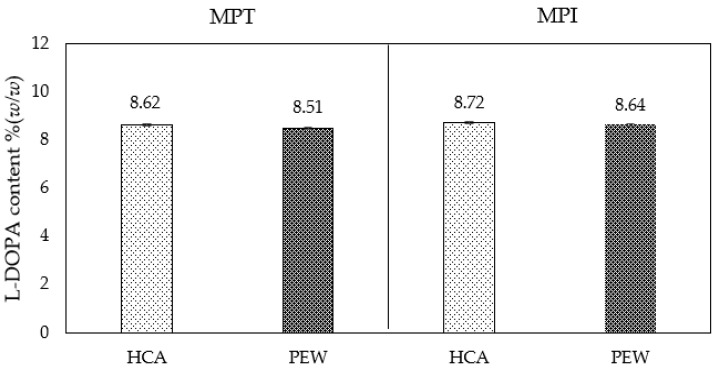
L-DOPA content of Thai and Indian MP varieties extracted to HCA compared with PEW. There was no statistically significant difference between extraction solvents.

The results showed that PEW can extract L-DOPA from both varieties. L-DOPA in PEW extracted MP values between 8.51 and 8.64% *w*/*w* in MPT and MPI, respectively (Figure 6). Moreover, extraction by PEW gave an insignificant result compared with that of conventional HCA. This confirmed and proved that the extraction system can be used to produce material with different characteristics. Since there were no differences between the two varieties, MPI was chosen for the physicochemical stability test because of its higher yield, low cost, and easier to harvest than the MPT variety.

### 2.4. Physicochemical Stability Test

#### 2.4.1. Color

The prominent physical property of the dried extract is indicated by the change in color. The color of the extracts was shown in Table 1. The initial experiment showed that the brightness of all extracts had a lightness value (L*) between 91.5999 and 96.4417. When the samples were stored under the acceleration condition after 6 and 12 months, their color was changed. The brightness was decreased in all samples. Interestingly, the samples extracted with PEW had the highest residual lightness value (L*) at 70.3645 followed by HCA (L = 60.0908), ACA (L = 57.1764), and CTA (L = 45.8105), respectively. After 12 months of storage, all extracts changed color into a dark brown shade except PEW-MP, which remained the lightest (L = 55.7375). These results showed that the extract of MP seeds with PEW retained the color of the extract mainly compared to other samples extracted with a single acid. It may be due to other organic compounds (Ellagic acid, Gallic acid; Emblicanin A & B, Phyllembein, Quercetin, and Ascorbic acid) in PEW that have the ability to inhibit the oxidation conversion reaction [31,32]. This is similar to a report that the addition of ginger combination extract improved the antioxidant properties and stability of avocado powder [33]. Among all acidifying agents tested, ACA was the worst in protecting color stability. It would be due to the instability of ascorbic acid in the solution during the extraction process, where heat was applied and a long extraction time caused its degradation [34]. In addition, the color of the extract can be easily changed by moisture in the environment [35]. Therefore, the dried extract must be kept in a tightly sealed container.

#### 2.4.2. pH in Extract Solutions

The pH value is another indicator of the stability of the extract because L-DOPA is known to be stable under acidic conditions. In general, the extract must maintain optimal conditions with a specific pH value. For L-DOPA, the optimal pH to maintain the condition is in the range of 2.0–4.0 [36]. This experiment controlled the acidity of the solvents at pH 3 before mixing with MP because it was observed in the previous study that the extraction condition of MP seed at pH 3 increased the extraction efficiency [37]. It is important to note that L-DOPA has zwitterionic (ion exchange properties). Therefore, its chemical form can be changed at the hydrogen atoms in its structure according to the pH value, resulting in a low solubility in water and alcohol at pH between 2.3 (pK_a1_) and 8.11 (pK_a2_) [13,38]. Moreover, it dissolves well in the acidic range, where the acidic pH in reducing oxidation as well [39]. Therefore, acidity is important in maintaining the stability of the L-DOPA extract.

**Figure 7:**
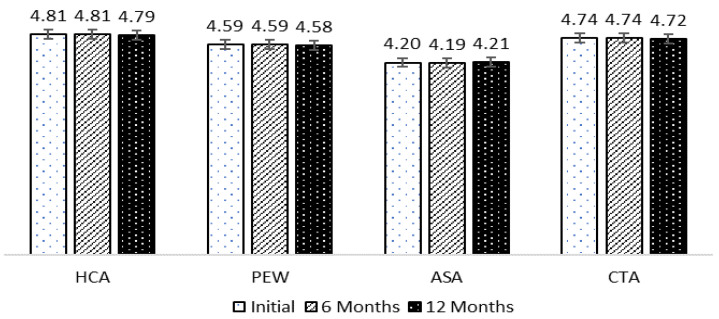
The stability of the pH value in MP extracted with various solvents. It is not significant by Duncan’s multiple range test.

The pH of each solution slightly increased after extraction between 4.20 and 4.81. After storage and dissolving in water to test the pH, each sample changed only slightly, even at 6 and 12 months, without significant difference (Figure 7). In this work, PEW was used because it contains natural acids. Although PEW was prepared at a concentration of 0.25–20.00%, the pH of the solution was still in a range of 4.02–4.96, as shown in Figure 2. It is possible that further adjustment of pH with other acids to lower the pH below 2.3 may help increase the solubility of L-DOPA.

### 2.5. Stability of L-DOPA in Extracts

**Figure 8:**
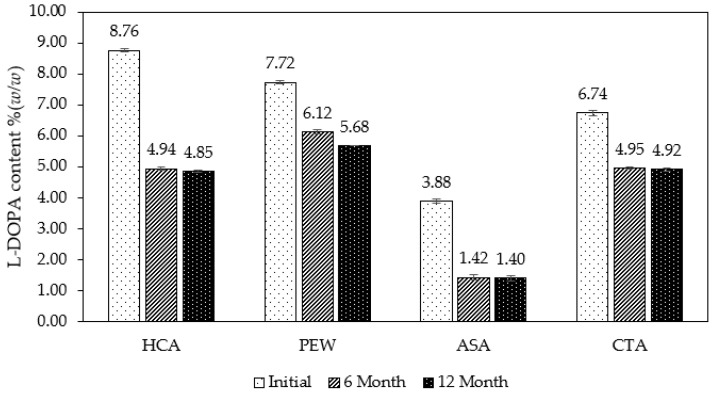
L-DOPA content in various extraction solvents observed during the stability test. There were three-time points: initial stage (Day 0), 6 months, and 12 months. The result was shown as a mean value, and S.D. was indicated as a bar (*n* = 3).

L-DOPA in the extract can be degraded over time. In the accelerated condition, the L-DOPA content in the extract powder determined by HPLC (Figure 8) was found to show a decrease in L-DOPA content over time, as expected. At the initial stage (Day 0), L-DOPA in the extract using HCA is the highest at 8.76% *w*/*w* followed by PEW (7.72% *w*/*w*), CTA (6.74% *w*/*w*), and ASA (3.88% *w*/*w*). However, the least amount of L-DOPA obtained in the ASA extract may be due to the fact that the preparation of ASA in solution was prone to degradation faster and higher than other solvents during the extraction process with MP seed powder [40]. Therefore, ASA cannot protect the degradation of L-DOPA, leading to a significant loss of extraction yield.

**Figure 9:**
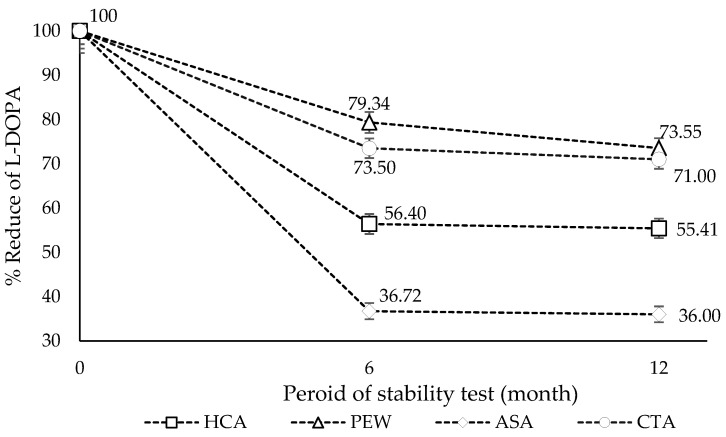
The percent of L-DOPA content remained in the extracts compared to the initial stage. The result was shown as a mean value, and S.D. was indicated as a bar (*n* = 3).

The stability of L-DOPA was observed after being kept for 6 and 12 months in the accelerated condition. The accelerated condition at 40 °C ± 2 °C/75% RH ± 5% RH and the minimum time period covered by data at submission 6 months used in this study refers to the International Council for Harmonization of Technical Requirements for Pharmaceuticals for Human Use (ICH) [41]. All samples were kept under this controlled condition. L-DOPA in all samples decreased largely after 6 months and 12 months, regardless of the solvent used, as shown in Figure 8. The reduction in L-DOPA appeared to be high for the first 6 months and then remained insignificant change between 6 and 12 months. When looking at the percentage of L-DOPA remaining in the extracts after 6 months from the initial stage, it showed the highest in the PEW extract (79.34%), followed by CTA, HCA, and ASA, respectively (Figure 9).

**Figure 10:**
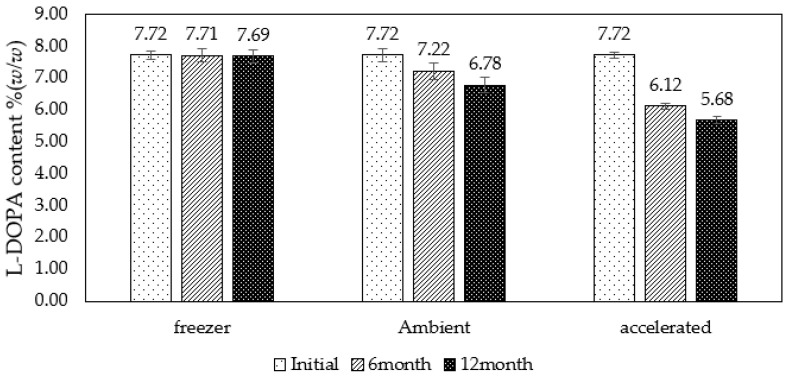
Comparison of different storage conditions for remaining L-DOPA content (%) of MP extract in the addition of PEW. The result was shown as a mean value, and S.D. was indicated as a bar (*n* = 3).

However, since accelerated storage for the worst-case study and extreme environmental conditions can lead to a rapid decrease in % L-DOPA, the PEW-containing extracts were compared with different storage conditions: freezer (−4 ± 2 °C), ambient of Bangkok Thailand (28 ± 2 °C) and accelerated (40 ± 2 °C), for information on extending the shelf life of L-DOPA, extracted with PEW (Figure 10). It was found that storage in the freezer was found to help maintain the best extract condition without chemical and physical changes. Meanwhile, ambient storage showed a slight decrease in L-DOPA even after 12 months of storage. Although the perfect storage condition would be in the freezer for maintaining the L-DOPA content, ambient storage reduces the content by less than 10% (Figure 10). The information in storage guidelines of the international program on Chemical Safety (IPCS) recommends storing in a dry place and avoiding exposure to excessive heat and light and in airtight containers at a temperature not exceeding 40 °C [42]. The results of this study can therefore support storage recommendations for preservation.

From the stability test results, PEW showed a good trend and contributed to the preservation of the physicochemical values of the extracts, probably because PEW contains important bioactive compounds that help reduce oxidation reactions. PEW has many benefit case studies. Chansriniyom et al., (2018) combined PEW and *Alpinia galanga* extracts in a 7: 3 ratio, and showed their protective effect on H_2_O_2_-induced oxidative stress in endothelial cells and its inhibitory effect on lipid peroxidation [43]. PEW was used as a food preservative in the study of Verma et al., (2021). PEW 2% was applied to increase protection against oxidation in goat nuggets while stored in the refrigerator to replace the antioxidation preservative (butylated hydroxytoluene). It resulted in good stability and quality of the nuggets [44]. In cosmetic research, Pereira and Mallya (2015) used PEW as a skin care ingredient for photoprotection to prevent oxidative stress caused by prolonged exposure to UV. PEW extract provides antioxidant activity and showed stability even after three months [45]. PEW was even used in biofuel research, as reported by Singh et al., (2019). PEW was used to reduce oxidation in biodiesel produced from jatropha and Pongamia as a replacement for costly and toxic synthetic oxidation inhibitors [31].

With many benefits of PEW, this study added to the evidence that PEW could be a solvent to extract other herbs. It provides good extraction results and maintains the quality of the MP extract. Furthermore, it was found that PEW extract alone contributed to the inhibition of the causative activity of Parkinson’s disease in the experimental rat [16]. Thus, it is expected that the combination of MP extracts with PEW would enhance the therapeutic effect as well. This could lead to further studies to determine its effectiveness to promote the treatment for Parkinson’s patients.

## 3. Materials and Methods

### 3.1. Preparation of MP Seeds

The MP seeds were purchased from a local market in Bangkok, Thailand, and collected at room temperature. Then, they were heated in a household microwave oven (TOSHIBA Microwave 700 W series MWP-MM20P(WH)) for 10 min until their seed coat was broken. The seeds were then finely ground and used for testing.

### 3.2. Chemicals

Ascorbic acid (99.5% assay) was purchased from Chemipan Corporation Co., Ltd. (Bangkok, Thailand). *Phyllanthus emblica* extract was purchased from 100% organic Gourmazia from India. Citric acid 1-hydrate 99.5% was purchased from Elago Enterprises Pty Ltd. (New South Wales, Australia). L-DOPA reference standard (3,4-Dihydroxy-L-phenylalanine), >99.9% from USP (Rockville, MD, USA). All solvents were analytical grade, including ethyl formate 98% and toluene 99.5% (Loba Chemie™, Tarapur, India), formic acid 98% (Fisher Chemical™, Loughborough, UK), and methanol 99.9% (RCI Labscan^TM^, Bangkok, Thailand).

### 3.3. Extraction Methods

15 g of seed powder was extracted with 150 mL of different acidified agents (pH = 3) that were hydrochloric acid, ascorbic acid, citric acid, and *P. emblica* water (PEW). The abbreviation is defined in Table 2. All samples were extracted by sonication for approximately 30 min at 70 °C. The extract was centrifuged to collect the supernatant and evaporated under a vacuum. Finally, the extract powder was collected in the vacuum bag for testing.

### 3.4. Qualitative Analysis by High-Performance Thin-Layer Chromatography (HPTLC)

Chemical Profile Fingerprint Identification test following a standard operating protocol of the International Association for the Advancement of High-Performance Thin Layer Chromatography [46]. Then, the samples (2 μL) were applied on plates of HPTLC silica gel (60 F254, 20 × 10 cm) (Merck, Germany) using Camag Linomat5 (Camag, Basel, Switzerland). The developing system was ethyl formate, toluene, and formic acid, with a water ratio of 30: 1.5: 4: 3 (*v*/*v*/*v*/*v*), and the running distance was 85 mm. The NP-PEG reagent was then used for derivatization. The plates were documented for the chemical profile under UV 366 nm by the Visualizer II using Vision CATS software (Camag, Basel, Switzerland).

### 3.5. Quantitative Analysis by HPLC

The method for quantitative L-DOPA by HPLC was modified from the report of Duan et al., (2021) [47]. The sample extracts and standard L-DOPA were dissolved in 0.1% (*v*/*v*) formic acid. The standard L-DOPA was 200–1000 ng/μL. The samples were then analyzed by HPLC (Agilent HPLC 1260 Infinity II, Agilent Technologies, Santa Clara, CA, USA) using a column (LiChrospher® 100 RP-18 5 μm LiChroCART® 250-4 HPLC cartridge, Merck KGaA, Darmstadt, Germany). Mobile phase A was assigned to 0.1% (*v*/*v*) formic acid in the water, and phase B was methanol by gradient programmed separation as follows: 1% B at 0 min to 10 min and 1–4% B from 10 to 20 min with a flow rate of 0.5 mL/min. The detection was measured at a wavelength of 282 nm. That method validation to confirm the effectiveness of the method followed based on ICH guidelines [48]. The calculated LOD and LOQ data were 0.23 and 0.69 µg mL^−1^, respectively.

### 3.6. Physicochemical Stability Test

The dried extract of MP seeds from the optimal extraction process was placed in a zip-lock bag and kept in accelerated condition at 40 °C ± 2 °C and relative humidity of 75% ± 5%. All samples were observed at 0, 6, and 12 months. Physical and chemical properties, including color, water pH dissolution, and L-DOPA amount, were recorded. The color quality of the extracts was determined by comparing the color charts of the color grab application range between light brown to a fine brown powder and was reported in the CIE L * a * b value system. The acidity of the extract was measured as the pH value (SevenCompact™ pH/Ion meter S220, Mettler-Toledo AG, Zürich, Switzerland). Some of the extracts were stored in a −4 °C freezer for analysis of L-DOPA content compared to accelerated.

### 3.7. Statistical Analysis

Analysis of variance one-way ANOVA was used. The significance of the differences was selected from Duncan’s multiple range test comparison tests. In the data, *p* < 0.05 was significant.

## 4. Conclusions

The extraction of L-DOPA from *Mucuna pruriens* seed with *Phyllanthus emblica* water yields good results in both physical and chemical properties. The color of the extract changed slightly, its pH was stable, and PEW did not react with L-DOPA. Moreover, there was a higher percentage of L-DOPA content compared to the control group, which is a hydrochloric acid solution. Therefore, our study proved that the improved extraction method using PEW could be used as a promising method to extract L-DOPA from MP seeds that could improve stability, avoid chemical solvents, and yet would also combine the benefit of PEW for the treatment of Parkinson’s disease. Finally, the extract could serve directly as a raw material in the production of herb products at the industrial level.

## 5. Patents

A petty patent was drafted and submitted to the Department of Intellectual Property, Thailand.

## Figures and Tables

**Table 1:** The color characteristics of the dried MP extracts with different solvents when stored in the accelerated condition at the beginning (day 0), 6 months, and 12 months later.

Sample	Day 0					6 Months					12 Months				
	CIE-Lab					CIE-Lab					CIE-Lab				
	(L)	(a)	(b)	Color		(L)	(a)	(b)	Color		(L)	(a)	(b)	Color	
HCA	94.7735	−3.1216	28.4629	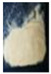		60.0908	17.8372	57.7983	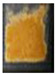		33.2482	8.7708	31.9524	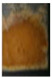	
PEW	96.4417	−7.2496	33.2448	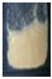		70.3645	5.3563	32.0316	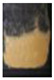		55.7375	9.5249	37.9486	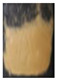	
ACA	91.5999	1.4565	14.1484	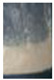		57.1764	13.1307	48.6883	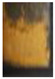		6.4405	14.2627	9.5094	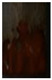	
CTA	91.7986	−0.4415	26.3880	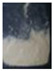		45.8105	4.8658	38.8984	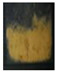		26.1554	13.5489	30.6758	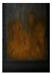	

**Table 2:** Abbreviation code of the acidified agent for the extract.

Acidified Agent	Chemical Structure	Abbreviation Code
Hydrochloric acid	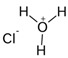	HCA
Citric acid	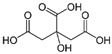	CTA
Ascorbic acid	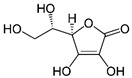	ASA
PE water	N/A	PEW

## Data Availability

Data is contained within the article.

## Sample Availability

Samples of the compounds are not available from the authors.
